# Supplementary material for: Identification of photoperiod-induced specific miRNAs in the adrenal glands of Sunite sheep (Ovis aries)
Source: Front Vet Sci. 2022 Jul 22;9:888207. doi: 10.3389/fvets.2022.888207 (PMC9354845; doi:10.3389/fvets.2022.888207)
Supplement: Supplementary file 1 [file Data_Sheet_1.ZIP › Supplementary file/miRDeep2 file.zip!/miRDeep2 file/Know_Structure/SPLP42A1/oar-mir-487a.pdf]

miRBase precursor : oar-mir-487a
Total read count : 105
oar-miR-487a-5p read count : 22
oar-miR-487a-3p read count : 82
remaining reads : 1

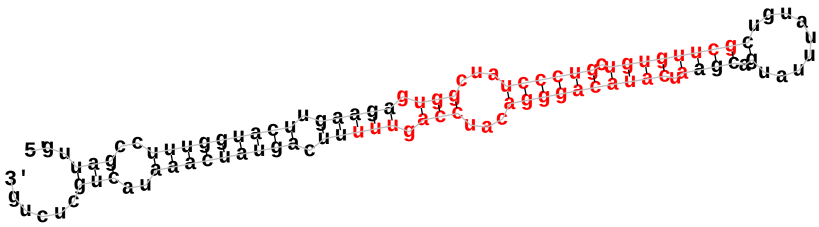

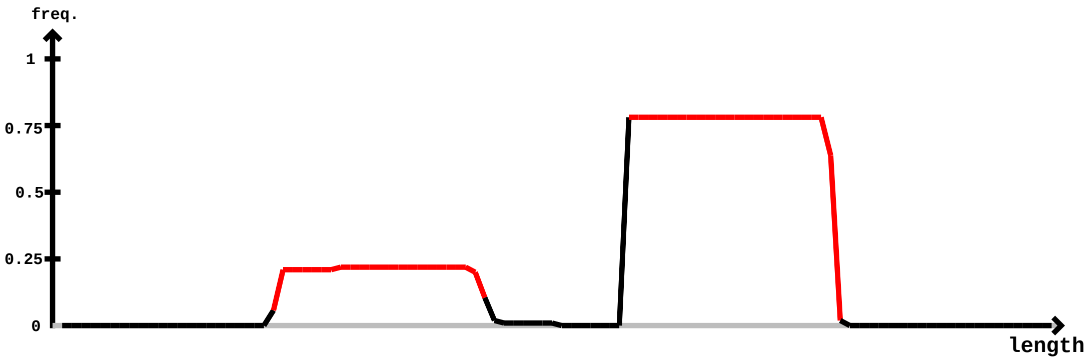

oar-miR-487a-3p
oar-miR-487a-5p

```
5'- guuagccuuugguacuugaagaguggcuaucccugcuguguucgcuguauuuaugacgaaucauacagggacauccaguuuuucaguaucaaauacugcucug   -3'   exp
    ..(((..(((((((((.(((((.(((...((((((.(((((((((.........).)))).))))))))))...))).))))).)))))))))..))).....   reads   mm   sample
    .....................aguggcuaucccugcuguguu.............................................................   1       0    seq
    .....................aguggcuaucccugcuguguuc............................................................   5       0    seq
    ......................guggcuaucccugcuguguu.............................................................   1       0    seq
    ......................guggcuaucccugcuguguuc............................................................   5       0    seq
    ......................guggcuaucccugcuguguucg...........................................................   9       0    seq
    ......................guggcuaucccugcuguguucgc..........................................................   1       0    seq
    ............................aucccugcuguguucgcuguauu....................................................   1       0    seq
    ..........................................................aaucauacagggacauccagu........................   15      0    seq
    ..........................................................aaucauacagggacauccaguu.......................   65      0    seq
    ..........................................................aaucauacagggacauccaguuu......................   2       0    seq
```
